# Infra-Low-Frequency Neurofeedback Treatment in Dysthymia: A Case Study

**DOI:** 10.3390/bs13090711

**Published:** 2023-08-28

**Authors:** Reinhard Tschiesner

**Affiliations:** Faculty of Education, Free University of Bozen/Bolzano, 39042 Brixen, Italy; reinhard.tschiesner@unibz.it

**Keywords:** neurofeedback, infra-low frequency, depression, dysthymia

## Abstract

Depression is one of the most common mental disorders worldwide. Dysthymia, a long-lasting form of depressive disorder that is also known as persistent depressive disorder (PDD) with pure dysthymic syndrome according to the *Diagnostical and Statical Manual of Mental Disorders* (DSM-5), is characterised by being difficult to treat. The most prominent therapeutic approaches in treating dysthymia are pharmacotherapy and psychotherapy, but recent studies also demonstrate the success of neurofeedback in treating individuals with depressive disorders. However, infra-low-frequency (ILF) neurofeedback, the main new neurofeedback protocol, lacks empirical evidence, and there is no evidence that it can treat dysthymia. This case report investigates the ILF neurofeedback method in a male patient with dysthymia. After 45 sessions of ILF neurofeedback combined with ILF synchrony, a decrease in symptom severity was found on assessment after treatment, and these results remained consistent at a low level at a 6-month follow-up. Additionally, the patient reported benefits on interpersonal and cognitive levels and in daily life situations. This study should incentivise further investigations into using ILF neurofeedback to treat dysthymia and all variations of depressive disorders.

## 1. Introduction

Depression has historically been one of the most common mental disorders worldwide [1]. In 2019, 7.2 per cent of EU citizens (about 37 million people) reported suffering from chronic depression [2].

In a clinical sense, depression is a serious illness that must be treated since it negatively affects the patient’s quality of life and their social, physical, and mental performance. Depression may be accompanied by suicidality [1,3,4].

Depressive disorders can be multifaceted. Dysthymia, for example, is a long-standing, often chronic, and usually fluctuating depressive disorder that does not have as many severe symptoms as a major depressive episode. Clinicians often overlook this because they have difficulty distinguishing it from a premorbid personality [5]. When a patient has dysthymia, however, they can go through phases of exhibiting the symptoms of a major depressive episode before remitting back to dysthymia. This disorder is referred to as “double depression”, composed of dysthymia and one or more episodes of major depression that overlap. For individuals with chronic depression who have never met the criteria of a major depressive episode or disorder in the past two years, the specifier “with pure dysthymic syndrome” is used. Dysthymia typically begins in early adulthood or after a bereavement [6]. Several studies have shown that in chronic depression, mood disorders are higher in first-degree relatives than in people with a nonchronic major depressive episode [7].

In the current *Diagnostical and Statistical Manual of Mental Disorders* (DSM-5) [8], dysthymia is classified as a “persistent depressive disorder” (code 300.4). Persistent depressive disorder (PDD) is a depression that lasts for at least half of all days for the duration of two years. However, a normal mood may occur for a few days or weeks between depressive periods. For a diagnosis of persistent depressive disorder, at least two of the following six criteria must be met: loss of appetite or overeating, insomnia or hypersomnia, low energy level or fatigue, low self-esteem, poor concentration, difficulty making decisions or hopelessness. These symptoms must not remit for more than two months and must cause marked distress or impairment. Furthermore, there are exclusion criteria for PDD: subjects must have never suffered from a manic or hypomanic episode, and symptoms should not be better explained by a non-mood psychotic disorder that is not caused by a physiological effect of a substance or other medical condition [8]. In the DSM-5, persistent depressive disorder is conceptualised as a broad diagnostic category encompassing a variety of manifestations, including chronic depression, persistent major depressive episodes, and dysthymia. An episode of persistent major depression consists of more severe symptoms than dysthymia [9]. Schramm et al. [5], therefore, recommend using the specifiers provided in the DSM-5 to clearly distinguish between the various forms of PDD: “persistent major depression episodes”, “intermittent major depressive episodes, with the current episode”, “intermittent major depressive episodes without the current episode”, and “with pure dysthymic syndrome”. Most people who suffer from pure dysthymia often subsequently experience a major depressive episode, underscoring how closely related dysthymia and chronic major depression are and that dysthymia cannot be considered just a mild form of depressive disorder [10]. In fact, the *International Statistical Classification of Diseases and Related Health Problems* 11th version (ICD-11) [11] did not introduce the concept of persistent depressive disorder (PDD), instead continuing to adopt the diagnostic category of dysthymic disorder. Major depressive episodes are categorised with a separate diagnosis. The ICD-11 working group considered this approach because changes over time can be mapped more precisely and are more easily differentiated [12].

In general, depressive disorders are treated with pharmacotherapy and psychotherapy [6,13] as well as (maintenance) electroconvulsive therapy (mECT) [13]. Most guidelines for treating depressive disorders cite cognitive–behavioural psychotherapy (CBT) as the most effective psychotherapeutic approach [6,13]. However, many studies also provide evidence for the effectiveness of other psychotherapeutic methods, such as psychodynamic, psychoanalytic, or systemic therapies. In addition to pharmacotherapy and psychotherapy as well as mECT, the guidelines also mention transcranial magnetic stimulation (TMS), although this is supported by a lower level of evidence [6,13]. Beyond these treatment recommendations, some studies indicate the efficacy of methods such as neurofeedback or electroencephalography (EEG)/fMRI-based biofeedback [14]. Indeed, recently Melnikov [14] identified at least 25 studies examining the effects of biofeedback and neurofeedback approaches in clinical trials as adjunct treatments for clinical depression. In a review, Larsen and Shirlin [15] provide evidence that neurofeedback can be considered “possibly effective” for treating depression (adapted from La Vaque et al. [16]). Shaffer and Zerr [17] consider frontal alpha asymmetry-based neurofeedback protocols and real-time fMRI neurofeedback to be “effective”, while Grin-Yatsenko et al. [18] found infra-low-frequency neurofeedback to be effective in three case studies with depressive people. Neurofeedback is generally a computer-assisted training method in which usually imperceptible parameters of the patient’s brain activity are selectively made perceptible to the patient via audiovisual and/or tactile feedback. Neurofeedback aims to influence a particular brain activity, which is then recorded via EEG [19].

The current literature identifies several neuromarkers for depressive syndromes. Grin-Yatsenko et al. [18] provided an overview of neuromarkers in the EEG activity of patients with depressive syndromes: most strikingly, they note the asymmetry in the alpha band, evidenced by increased alpha power in the left frontal region and/or the right parietal–temporal region [20]. Bruder et al. [21] compared the resting EEG of patients with anxiety disorder and patients with depression and anxiety disorder and found that the alpha asymmetry of subjects with depression and anxiety disorder differed from those with only anxiety disorder. Alpha asymmetry in non-anxious depressed subjects is characterised by less activation in the right than in the left posterior areas. In contrast, in anxious and depressed subjects, activation was higher in both the right and left posterior and anterior areas. Furthermore, other authors found bilaterally increased frontal alpha activity in patients suffering from major depression or other mood disorders [22,23]. There was also evidence of increased slow-wave activity in both the right [24] and left hemispheres [25].

In the 1960s and 1970s, before the discovery of these EEG neuromarkers for depression, the basics of neurofeedback therapy were discovered. Kamiya [26] and Sterman and Friar [27] used a system that measures brain waves and provides feedback in a visual and/or auditory way, thus reinforcing the occurrence of certain brain waves, and noticed that brain waves could change their activity. Most research on neurofeedback has focused on frequency bands from 8 to 25 Hz. In contrast, Birbaumer [28] studied slow potential shifts related to motor and cognitive preprocesses at frequencies below 0.1 Hz and developed the slow cortical potential (SCP) neurofeedback protocol. Frequencies below 0.1 Hz reflect fluctuations in cortical excitability, and neurofeedback that is training these frequencies aims to act on this tonic central arousal. In 1996, an optimal response frequency was established, which is very similar to the idea of an optimal level of arousal and sensation seeking [29,30,31,32], across the broader EEG spectrum. The individualisation of an optimal response frequency led to better outcomes in neurofeedback sessions and the development of neurofeedback in the infra-low-frequency area. Infra-low-frequency (ILF) neurofeedback is a further development of SCP neurofeedback. In ILF neurofeedback, frequencies between 0.0001 and 100 mHz are applied [33,34]. A unique feature of ILF neurofeedback is the implicit processing of the feedback signal, which presumably modulates neuronal networks involved in brain self-regulation [35].

Empirical evidence for treating affective disorders and particularly depressive dis-orders with ILF neurofeedback is still poorly explored. Only Grin-Yatsenko et al. [18] have published three case reports of depression in which ILF neurofeedback was performed. After 20 ILF neurofeedback sessions lasting 30 to 45 min each, the outcome was measured with clinical rating scales and quantitative EEG (QEEG) measures before, after, and in the follow-up of the intervention. Although the ILF neurofeedback protocol does not generally directly improve frontal alpha asymmetry or suppress theta activity, the researchers found significant improvement in all patients’ EEG spectra and depression scales after ILF neurofeedback training. Theta power decreased in all three patients, and alpha activity in the QEEG decreased in those with high alpha activity in the QEEG. None of the three patients showed signs of clinical depression at the end of the treatment or one year after the neurofeedback sessions.

It is still unclear whether ILF neurofeedback also works in persistent depressive disorder (PDD) or dysthymia. Schramm et al. [5] concluded in a review that treating dysthymia is generally more complex than treating a major depressive disorder. In PDD and dysthymia, pharmacological therapy or psychotherapy or both combined are generally the first treatment choice, but they are less effective for these disorders than in nonchronic forms of depressive disorders [36,37]. Cuijepers et al. [36] found that the effect of psychotherapy, although significant, was small. When patients had specific characteristics that are risk factors for chronic depression, such as childhood maltreatment, developmental trauma, or early childhood neglect, therapeutic success was often better due to specific psychotherapeutic approaches [37]. However, approximately 40 per cent of patients with PDD are treatment-resistant [38]. Pharmacotherapy with serotonin-reuptake inhibitors (SSRIs) was more effective than placebo, while treatments with fluoxetine and with imipramine were less effective than other medications [39]. For pure dysthymia, pharmacotherapeutic treatments still seem to be more effective than psychotherapy [5].

The generally lower treatment success for people with PDD or dysthymia may be due to several reasons: first of all, treatment delay. Often, individuals do not seek help for years. This delay in starting an intervention leads to a less positive treatment outcome. Second, patients with PDD or dysthymia show low motivation for therapy as they often believe that their dysphoria is part of their personality and not treatable. In addition, their pessimistic thinking leads to passivity and reduces therapeutic motivation [5].

Since the spontaneous remissions of PDD or dysthymia are rare [40] and the mental illness is distressing to the patient, it is important to inspect how to treat PDD and dysthymia more effectively. Therefore, this study aims to investigate whether a new form of treatment could lead to promising effects. More precisely, it aims to investigate whether ILF neurofeedback could be an appropriate method for treating PDD or dysthymia without comorbid disorders. Currently, there is no empirical evidence for ILF neurofeedback treatment in PDD or dysthymia, and overall, there is a need for further outcome research on the effect of ILF neurofeedback since it is a relatively new training method.

This study used a single case study with a pre–post follow-up design to analyse the effects of ILF neurofeedback therapy on dysthymia/PDD in which traditional therapeutic approaches are less effective. In particular, the following research questions are addressed. How does ILF neurofeedback therapy affect a person with dysthymia? Is it possible to significantly reduce the patient’s symptoms of dysthymia, and does this improvement have a long-term effect? Does ILF neurofeedback therapy also affect somatic accessory symptoms that are not typical of PDD or dysthymia but accompany it, as well as cognitive abilities and interpersonal behaviour? Is a patient with dysthymia continuously motivated to attend ILF neurofeedback sessions over several months?

## 2. Materials and Methods

### 2.1. Case Study Design

The procedure of the study can be described as follows. After recruiting a patient with dysthymia, a pre-intervention assessment takes place. The patients’ reactions were recorded in a report written after each session during the intervention. The second assessment occurred at the end of the ILF neurofeedback intervention. To test the stability of the effects, a follow-up was conducted 6 months after the end of the intervention. Overall, the investigation took approximately one year.

### 2.2. Participant Description

The case study was conducted with a man aged around 30. This study will refer to him using the pseudonym “Matt”. Matt is employed in public administration and is in a long-term relationship. His mother died when he was in primary school, and he grew up with his father, sibling, and grandparents. In his family, a second-degree relative suffers from a severe major depressive disorder with psychotic symptoms.

Matt decided to try neurofeedback after doing four hours of psychiatric counselling and six hours of psychological counselling for his depressed mood in an autumn–winter period. He was not taking any medication and had never received psychotherapeutic treatment. His primary care physician confirmed in a recent blood test that he had no thyroid gland or other physiological diseases that could have influenced his mood. His psychologist recommended neurofeedback treatment to him.

In the first meeting, Matt explained that he had experienced depressed mood and dysphoria since his youth, which he remembered as a grey period. He described being passive and noted that he could not initiate actions independently. He reported spending whole afternoons observing people without engaging with them. Currently, Matt finds it difficult to assert himself and defend his point of view at work. Additionally, he is very concerned about the future and worries that he cannot influence his life as he lacks control. He also said that he thinks he is clumsy and incompetent. Small daily arguments with others can put him in a deep sad rigidity, which lasts for several days. He also finds it difficult to plan activities and focus on required actions and further reported severe mind wandering, which he said was unrelated to his mood. He could not focus on lessons at school when he was younger and now struggles to focus on important work tasks. He also said that he used to fall asleep when learning for class. Being unable to hold on to thoughts bothers him a lot. He also finds it difficult to motivate himself to do things.

Generally, his condition gets worse with the change from winter to summer and vice versa. In spring and autumn, his distress is most pronounced. In the first meeting, which was in spring, he felt exhausted. Suicidal ideation was not present. He hoped that the neurofeedback treatment would ‘cure’ his rumination, mind wandering, and inhibited behaviour. Additionally, he wanted to improve his accuracy in accomplishing tasks and his organisation of daily activities, which he described as chaotic.

### 2.3. Clinical Assessment: Instruments

All instruments and questionnaires were completed by a qualified psychologist before the first meeting with the neurofeedback therapist took place. Instruments and questionnaires were completed again at the end of the neurofeedback intervention and 6 months after the intervention to check the effects’ stability. The Structured Clinical Interview for the DSM-5 (SCID) and the QikTest were conducted only at the beginning and end of neurofeedback treatment.

#### 2.3.1. Beck Depression Inventory II (BDI-II)

The BDI-II is a self-reporting instrument that uses 21 items to assess the symptom severity of depression. The inventory is an operationalisation of the depression criteria of the DSM-IV-TR. The Cronbach alpha of the inventory is between 0.89 to 0.93. Processing time is 5 to 10 min [41,42]. Scores between 0 and 12 BDI points indicate minimal depression, 13 to 19 indicate mild depression, 20 to 28 indicate moderate depression, and 29 to 63 indicate severe depression [42].

#### 2.3.2. Brief Symptom Checklist (BSCL)

The BSCL [43] is a self-reporting questionnaire composed of 53 items of the more extensive Symptom Checklist composed of the 90-item standard version (SCL-90-S) [44], which regards symptoms of mental disorders. Just like the SCL-90-S, the BSCL consists of a somatisation subscale, an interpersonal sensitivity subscale, an obsessive–compulsive subscale, a psychoticism subscale, a phobic anxiety subscale, a paranoid ideation subscale, a depression subscale, an anxiety subscale and an anger–hostility subscale. The Global Severity Index (GSI) indicates the current symptom load. The Positive Symptom Distress Index (PSDI) provides evidence of the intensity of symptoms, and the Positive Symptom Total (PST) indicates how many symptoms are associated with distress. For the subscales, in a sample of people in psychotherapeutic treatment, Cronbach’s alpha range is from 0.70 to 0.85, whereas for the GSI, an alpha of 0.95 is found [43].

#### 2.3.3. Structured Clinical Interview for DSM-5 Clinician Version/Personality Disorder (SCID-5-CV/PD)—German Version

The Structured Clinical Interview for the DSM-5 [45,46] is used to assess the most popular disorders of the DSM-5 [8], which are affective disorders, schizophrenia spectrum and other psychotic disorders, substance-related and addictive disorders, anxiety disorders, obsessive–compulsive disorders and trauma- and stress-related disorders, attention-deficit/hyperactivity disorder in adulthood, adjustment disorder, and others. The personality disorder element of the SCID-5 [46] allows the screening of cluster A, B, or C personality disorders according to the DSM-5. An SCID takes at least 45 min, but can last longer in subjects with a more complex history and symptoms. Reliability scores from 0.60 (agoraphobia) to 0.83 (specific phobia/social phobia) for clinical disorders were found. For the SCID-5-PD, interrater-reliability scores (kappa) from 0.78 (antisocial personality disorder) and 0.91 (paranoid personality disorder) were found. SCIDs are considered the ‘gold standard’ in assessing mental disorders [47].

#### 2.3.4. QikTest/Continuous Performance Test (CPT)

The QikTest is a handheld device that tests continuous visual attention independently of a computer. For 21 min, light signals are presented that the subject has to react to. After the test, individual results are compared with a database of around 20,000 subjects and a report generated automatically. The report gives a general performance index reflecting the speed and consistency of responses and an accuracy index indicating inattention or impulsivity of subjects, which means that subjects reacted before the stimulus appeared [48].

#### 2.3.5. Symptom-Tracking and Anamnesis

The symptom tracking tool is a catalogue of symptoms and disorders that are initially rated on a Likert scale from 0 (not present at all) to 10 (present to the full extent and very stressful) by a patient. After the patient’s rating, a clinician explains the symptoms and disorders and discusses the scores to obtain a consensus about the different scores and the symptom severity. Symptoms and disorders are grouped into categories of sleep, attention and learning behaviour, sensory symptoms and perception, behaviour, emotions, physical symptoms, and pain. The tool was developed by the EEG Expert company [49] and enables the tracking of neurofeedback therapy while revealing changes.

### 2.4. Treatment: Infra-Low-Frequency Neurofeedback

The therapist in this ILF neurofeedback treatment attended the ILF neurofeedback basic training course and the alpha–theta and synchrony neurofeedback course. The therapist has 15 years of experience as a psychologist and has used ILF neurofeedback protocol for some years.

Before the neurofeedback training starts, a treatment plan must be created with the help of a detailed anamnesis that examines the patient’s medical history, mental health, emotional states, habits, etc. An ILF neurofeedback training usually lasts for more than 20 sessions (at least 2 sessions per week) [50]. Electrodes are placed according to the 10–20 system. Generally, synchrony sessions occur within an ILF neurofeedback training and require the trainee to possess a certain stability and self-regulatory ability. Synchrony sessions are held alternately with (standard) ILF neurofeedback sessions. In the following sections, these two forms of intervention are described in more detail.

#### 2.4.1. Two-Channel ILF Neurofeedback Training

A characteristic of ILF neurofeedback is that the response frequency depends on the client and must be identified in the first sessions. The optimal response frequency, considered the optimal level of arousal, is associated with mental states like alertness, relaxation, and calm. High response frequencies would cause headaches, anxiety, or sleep disturbances, whereas low response frequencies lead to fatigue, apathy, sadness, or depressive symptoms. In both cases, the therapist must adjust the frequency [33,50,51]. Another characteristic of ILF neurofeedback is bipolar montages. These were newly introduced in the ILF neurofeedback protocols since training effects had a higher impact on trainees [51]. Additionally, the ILF neurofeedback training signal is deduced from two channels of the EEG. Where electrodes are placed depends on the patient’s symptoms. Placements in the anterior half of the brain are meaningful in output processing and executive functions, whereas placements in the posterior half are meaningful in input and sensory processing. Placements on the right side impact body and emotional awareness and are significant for feeling safe and calm, whereas placements on the left are involved in planning the future and in a detailed analysis of the patient’s environment. Following the protocol guide, placements were changed during an ILF neurofeedback treatment [33].

#### 2.4.2. Two-Channel ILF Neurofeedback Synchrony Training

Neurofeedback synchrony training was introduced by Fehmi, Adkins, and Lindsley [52] as they investigated the role of synchronised neural activity for the central nervous system in primates. The production of synchronised brain waves is associated with a particular form of effortless attention. This training protocol can be used for chronic stress-induced diseases. The reward frequency in those synchrony training protocols was 10 Hz, and electrodes were placed at Fpz, Cz, Oz, T3, or T4 positions [33,53]. When brain waves are syncing, the amplitude of their sum is higher, and the subject would be rewarded with a beep or a light flash and should increase synchrony. Othmer [33] introduced an ILF synchrony protocol that works with a reward frequency of 0.05 Hz next to a 10 Hz and 40 Hz synchrony protocol, which helps strengthen the core sense of self and has proven very tolerable to patients. Alpha synchrony or 10 Hz neurofeedback is indicated in the patient with a hyper-aroused nervous system that must be calmed (i.e., in anxiety disorders, obsessive–compulsive disorders), whereas 40 Hz or gamma synchrony neurofeedback could be used in people who show deficits in attention and need a calm focus.

Usually, in synchrony neurofeedback, Fpz and Pz placements are used as active electrodes, A1 and A2 as reference electrodes, and Cz is used as ground placement.

## 3. Results

### 3.1. Clinical Assessment Preintervention

In the Beck-Depression Inventory II (BDI-II) before starting with the neurofeedback therapy, the patient got a score of 13, which corresponds with “mild or moderate depressive symptoms” (under the clinical threshold). In the Continuous Performance Test (Qik CPT), Matt obtained a performance index of 108 (mean = 100; standard deviation = 15) and an accuracy index of 103 (mean = 100; standard deviation = 15). The BSCL reveals a Global Severity Index (GSI) of T = 62, which indicates a symptom load with clinical relevance and a “depression” score of T = 65, which indicates a clinically significant level of depression. The symptom tracking sheet reveals that the most significant symptoms were acute tiredness/fatigue with a score of 7 (1 lowest value; 10 highest value), chronic difficulty in making decisions with a score of 7, chronic worries and concerns with a score of 6, chronic untidiness with a score of 6, and chronic poor short-time memory with a score of 6 (see Table 1).

Before starting the neurofeedback treatment, the SCID-5-CV/PD [45,46] interview revealed that Matt from persistent depressive disorder (dysthymia, 300.4) with pure dysthymic syndrome in the last two years with mild symptom severity. There were no comorbid disorders. The SCID diagnosis aligned with the clinical diagnosis of two independent psychologists with PhDs in clinical psychology and more than 15 years of clinical experience.

### 3.2. ILF Neurofeedback Treatment: Goals, Plan and Process (Intervention)

The first objective of the ILF neurofeedback treatment was to reduce fatigue and symptoms related to PDD and dysthymia, particularly stabilising mood and reducing passivity. Alongside these objectives, cognitive functions such as taking decisions to establish control, maintaining concentration and focusing, and being more assertive with others should be improved. Additionally, self-esteem and assertiveness in interpersonal relationships should be increased.

According to the Othmer manual [33] of ILF neurofeedback, anamnesis, the results of questionnaires, and interviews revealed the necessity of calming the nervous system, which seems hyper-aroused. Calming also improves self-regulation, especially in patients with traumatic experiences [54], to avoid decompensation or worsening conditions. The appropriate electrode position is T8-P4 on the right hemisphere. After calming the nervous system and improving self-regulation, instabilities shall be treated using the interhemispheric T7-T8 electrode placement. After stabilising, problems with poor focus on activities and concentration should be treated with a prefrontal electrode placement T7-Fp1. Several ILF neurofeedback sessions and 6 to 8 2-channel ILF synchrony sessions were planned to improve self-esteem and assertiveness after training on basic electrode placements.

In the following, the course of the sessions is described.

#### 3.2.1. Sessions 1 to 5: Starting Placement and Search for Optimal Response Frequency

As planned, treatment started with T8-P4 electrode placements. The first session lasted 9 min. After the first session, Matt reported that he did not notice any abnormalities. Gradually, the duration of the sessions was increased. An optimal training frequency of 0.001 mHz was established. He reported boredom and restlessness during the sessions where a high response frequency (>0.001 mHz) was chosen and extreme tiredness that occurred like seizures at too-low response frequencies (<0.001 mHz). He explained that he felt he was about to fall asleep. He also said that he had to try hard to stay awake and alert during the sessions.

After the fourth session, he reported headaches, which he had not had for years, and that he had argued with his girlfriend. He said that he felt very irritable, which was unlike him, so electrode placements were changed to T7-T8 to stabilise his nervous system.

With the T7-T8 electrode placement, headaches and irritability disappeared. In the fifth session, the duration reached 18 min, but he reported tiredness after the session and weariness outside the neurofeedback training.

#### 3.2.2. Sessions 6 to 24: Treatment Duration of Sessions and Additional Placement

The duration of the neurofeedback sessions was reduced drastically to establish whether the long session duration caused the tiredness attacks. After cutting sessions to 6 min, tiredness during sessions decreased significantly. After this time reduction, the duration was slowly increased.

In the ninth session, the T7-Fp1 placement was added to the basic placement for 5 min. Before and after the 5 min training with the new placement, a T7-T8 session, took place to counter any side effects. In the tenth session, Matt reported needing to do sport for the first time. Additionally, he noticed that now he takes the initiative and puts plans into action. He also said that he enjoyed coming to the neurofeedback sessions.

In the following sessions, he continued reporting that he took the initiative now and did not procrastinate. Further, his girlfriend noted these differences in his behaviour. He reported that his mood was more stable. External problems did not throw him off track.

Towards the end of the sessions, independently of the electrode placement, he reported being overcome with tiredness. Adjustments in the ORF did not have an effect. Therefore, in session 17, instead of the previous T7-T8 after the T7-Fp1 placements, the calming electrode placement T8-P4 was added for 5 min. He immediately noted that tiredness changed into an enjoyable sense of warmth and ease. In the 19th session, an attempt was made to increase the duration by one minute for each placement. However, at 15 min, he began feeling tired. For this reason, the sessions were limited to 15 min.

#### 3.2.3. Sessions 25 to 45: Additional ILF Synchrony Training

In session 25, 2-channel ILF synchrony neurofeedback (reward frequency 0.05 Hz) was applied to improve Matt’s self-esteem. The first synchrony session lasted 10 min. Active electrodes were placed on the midline of the scalp (Fz and Pz). The midline placement of electrodes in synchrony-training is especially suitable for subjects with instabilities and should avoid such side effects as tiredness. After the first ILF synchrony session, 5 min of T7-T8 ILF neurofeedback was added to stabilise Matt’s nervous system, if necessary. Immediately after the first neurofeedback session, he did not report any side effects; therefore, the T7-T8 2-channel ILF neurofeedback could be dispensed with.

In the session after the first synchrony training, Matt reported that his condition after the synchrony training was quite special. He said he felt elated and that he could mentally observe what was happening from a distance. He felt elevated and noted that he now engaged with other people at eye level. From that point onwards, ILF and synchrony sessions were held alternately. Tiredness, which he described in the first sessions as a side effect of neurofeedback, did not reappear during and after session 25 or in any following sessions.

After session 32, the neurofeedback treatment was interrupted for a week due to the holidays. After it restarted, two ILF sessions were conducted before alternating synchrony sessions were conducted again. Those settings remained unchanged for the rest of the sessions.

### 3.3. Clinical Assessment Postintervention

At the end of the treatment, difficulties in decision-making decreased by 7 points, fatigue/tiredness decreased by 6 points, difficulty in thinking clearly decreased by 5 points, worries and concerns decreased by 5 points, bad social/emotional resonance decreased by 5 points, feeling unmotivated decreased by 4 points, untidiness decreased by 4 points, depressed mood decreased by 3 points, poor short-term memory decreased by 3 points, maintaining concentration decreased by 3 points, and difficulty organising activities decreased by 1 point in the symptom tracking.

The score of the BDI-II decreased from 13, which corresponded to mild or moderate depression, to a score of 3 after treatment, which corresponded to a minimal severity of depression.

The BSCL evidenced a Global Severity Index (GSI) T score of 40 and a depressiveness T score of 57, which are within the standard and do indicate any clinical burden of symptoms.

The Continuous Performance Test (Qik CPT) after the end of the treatment revealed a performance index of 102 (mean = 100; standard deviation = 15) and an accuracy index of 106 (mean = 100; standard deviation = 15). Therefore, the attentional performance remained unchanged.

The SCID interview did not give any indication of a mental disorder after treatment.

### 3.4. Clinical Assessment at 6-Month Follow-Up

Six months after the neurofeedback treatment on the symptom tracking (see Table 1 and Table 2), the posttreatment measure decreased from one to zero on fatigue/tiredness, from 0 to 1 for depressed mood, from 0 to 1 for difficulty in thinking clearly, and from 4 to 3 for difficulty in organising activities. Difficulty making decisions, bad social/emotional resonance, maintaining concentration, feeling unmotivated, worries and concerns, and untidiness did not change, whereas poor short-term memory increased from 3 to 4.

The Global Severity Index (BSCL) decreased from a T score of 40 to a T score of 34 and is now considered below-average symptom load.

The Beck Depression Inventory II score decreased from 3 to 1, which is considered a minimal depressive symptom load.

## 4. Discussion

The present findings can be considered an extension of Grin-Yatsenko et al.’s [18] case reports, which generally considered ILF neurofeedback to be helpful with a positive outcome for depression that remained consistent a year after beginning the treatment. In this case, the report’s focus was on a special form of depressive disorder, namely, the persistent depressive disorder with pure dysthymic syndrome.

The investigated patient met the diagnostic criteria of PDD with pure dysthymic syndrome according to the DSM-5. Further, symptomatic features mentioned by Schramm et al. [5] were noticed. The onset of depressive symptoms occurred early in Matt’s youth; this can be considered early. Another risk factor for the early onset of chronic depressive symptoms was Matt’s traumatic life event. Matt lost his mother when he was in primary school. Illustrating the typical treatment delay in patients suffering from chronic forms of depression, it could be stated that Matt never sought help before consulting the psychiatrist and the clinical psychologist two months before starting with neurofeedback. Thus, he lived with his symptoms for more than fifteen years and treatment started with a delay. Regarding the typical low therapy motivation of patients with chronic depression, Matt must have had a positive moment when he took the initiative to change. He noticed that his chronically depressed mood interfered with his life so much that he had to seek help. He did not demonstrate low therapy motivation; instead, he indicated that he enjoyed the experience. It must be noted that Matt cannot be considered as the typical patient with dysthymia. As Klein et al. [5] mentioned, people with dysthymia often also suffer from a comorbid major depression episode, and so experiences that result from this therapy are only relevant for patients with pure dysthymia without any comorbid disorders.

This investigation provides indications that after 45 neurofeedback sessions composed of ILF neurofeedback sessions, using T7-T8, T8-P4, and T7-Fp1 placements, and ILF synchrony neurofeedback sessions, Matt’s persistent depressive disorder (with pure dysthymic syndrome) remitted. The SCID did not reveal any symptoms of an affective disorder. Regarding the clinical assessment, the depression and symptom severity questionnaire scores decreased from clinically significant severity to a normal range. Further, the symptom tracking questionnaire, which includes physical symptoms and habits, indicated a decrease in symptom severity. The BDI-II should be highlighted here, as its quantitative statements about symptom severity of depressiveness are phenomenologically relevant and could detect an appearance of depressive disorder. Matt’s scores before and after the ILF neurofeedback intervention were under the threshold of a clinically significant depressive disorder. However, the scores had diminished after the end of the intervention. At the 6-month follow-up, the effects of the assessments remained consistently low and below the clinical threshold. Approximately a year passed from the first session until the follow-up assessment, similar to in Grin-Yatsenko et al. [18] investigation. Spontaneous remission is rather unlikely, as Matt’s symptoms have persisted for many years, and he is aware of the usual fluctuations in his mood.

Furthermore, it seems that the neurofeedback treatment process affected Matt’s executive functions, especially his planning and attention regulation, which increased after around 10 sessions. Regarding attention, on the one hand, only considering the QikTest, performance in attention did not change. However, the QikTest considered only visually focused attention [55,56]. Only testing Matt’s attention is an inappropriate method for investigating all facets of attention. On the other hand, Matt said that his attention improved in daily life activities and situations, which underlines the benefit of the neurofeedback intervention. During the two-channel ILF neurofeedback sessions, he reported that dysphoria and mood swings disappeared, whereas more self-esteem-related symptoms improved later with two-channel ILF synchrony neurofeedback sessions.

Cognitive and affective impairment caused by a disorder in most outcome research is usually operationalized by established instruments such as the BDI, the BSCL, and the SCID-5. However, these instruments are not able to reveal therapeutic changes at all levels. Especially subjective significant changes of the symptom burden, which are not mentioned in the standardized instruments, can be neglected [57]. This is attempted to be counteracted by additional ad hoc questions of the therapist. Nevertheless, such information does not have the same impact as that of a standardized and established instrument for outcome research.

Regarding the number of sessions, it is recognized that the higher the number of sessions that people with dysthymia have, the better their outcomes; this is particularly the case in psychotherapeutic interventions [5,58]. Further, the number of sessions in this treatment can be considered elevated. An ILF neurofeedback treatment typically lasts at least 20 sessions [50], but there is no empirical evidence of how many sessions are necessary for an effective ILF neurofeedback intervention. Strehl [59] mentioned that neurofeedback interventions independent of the protocol range between 25 sessions, the minimum for neurofeedback treatment, and the average length of a psychotherapeutic intervention corresponding to 40 sessions. In Grin-Yatsenko et al.’s [18] study and in a former study on SCP-neurofeedback in depression [60], the intervention lasted 20 sessions. In their review, Fernandez-Alvarez et al. [61] indicated that Dehghani-Arani et al. [62] offered 30 neurofeedback sensory motor rhythm training sessions to depressed subjects. However, in this study, depression was also considered a comorbid phenomenon in patients primarily with an opiate addiction. The number of 45 sessions in the presented case report is twice as high as in Grin-Yatsenkos et al.’s [18] study but is in the range of Strehl’s [59] estimation of the duration of a neurofeedback intervention. The number of sessions necessary for a patient’s positive outcome depends on their learning pace and modality and varies between individuals [59,63]. Consequently, the number of sessions cannot be considered too low. A higher number of treatment sessions can lead to reinforcement and stabilisation of treatment effects [58].

It should also be underlined that the two-channel ILF neurofeedback was not used as a monotherapy. With two-channel ILF neurofeedback, an improvement in executive functions could be seen, but symptoms that affected self-esteem remained unchanged. An improvement in self-esteem-associated symptoms was only noticeable with the introduction of the ILF synchrony neurofeedback protocol. The indications of an improvement in the patient’s clinical condition likely originate from the combination of two-channel ILF neurofeedback and ILF synchrony.

## 5. Conclusions and Future Work

This case report documents the first instance of a treatment of a patient suffering from dysthymia with ILF neurofeedback protocols (two-channel neurofeedback and ILF synchrony neurofeedback) under practice conditions. For the empirical evidence of ILF neurofeedback, ‘outcome research’ foresees the concepts of efficacy and effectiveness, which both have to demonstrate the benefit of ILF neurofeedback protocols. Generally, the design and implementation of randomized controlled trials (RCTs) is essential but not sufficient to demonstrate efficacy as well as effectiveness. RCTs focus on the efficacy of an intervention and their strength is a high internal validity. When planning such studies, an attempt is made to create a laboratory-like situation via various control techniques to be able to clearly attribute the effects to the therapeutic intervention. However, the weakness of RCTs is external validity. This is where the importance of effectiveness studies comes into play, highlighting the benefits of an intervention under real-life conditions. Effectiveness studies tolerate confounding variables to establish everyday conditions in order to assess the practicality and benefit of a method. For ILF neurofeedback in dysthymia, there are neither studies on efficacy nor on effectiveness. Findings of this case report deliver first indications for the effectiveness of ILF neurofeedback in dysthymia [57,64].

Outcome research generally does not investigate what would be restructured or changed through a therapeutic process. This is a typical task of ‘process research’, which, however, was not the focus of this case study, but could be considered in further research. The outcome-process study of Grin-Yatsenko et al. [18] investigated changes in EEG-frequency bands and event-related potentials (ERPs) before, after, and on follow-up of ILF neurofeedback treatment and detected changes in several neuromarkers in subjects with a depressive disorder. Other studies considering structural or functional changes induced by ILF neurofeedback in patients with a depressive disorder are not known. Regarding dysthymia, there are so far no indications about such changes through an ILF neurofeedback intervention at all. As such, in order to understand what happens during an ILF neurofeedback intervention, it is important to plan and conduct studies that investigate structural and functional changes through ILF neurofeedback in dysthymia, taking into account, e.g., functional magnetic resonance imaging (fMRI), as Dobrushina et al. [35] have done with healthy subjects, or using other neuroimaging methods, QEEG or ERPs.

In conclusion, it can be stated that the therapeutic success in Matt’s ILF neurofeedback treatment is a promising finding, showing the potential of this method in the treatment of dysthymia and that it is worth conducting more ample investigations into applying ILF neurofeedback focusing on efficacy and effectiveness as well as process research.

## Figures and Tables

**Table 1:** Symptom Tracking (pre- vs. postintervention vs. follow-up).

Symptoms		Symptom Severity (Preintervention)	Symptom Severity (Postintervention)	Symptom Severity (6M Follow-Up)
Fatigue/tiredness	acute	7	1	0
Difficulty making decisions	chronic	7	0	0
untidiness	chronic	6	2	2
Worries and concerns	chronic	6	1	1
Poor short-term memory	chronic	6	3	4
Difficulty thinking clearly	chronic	6	1	0
Difficulty in organising activities	chronic	5	4	3
Maintaining concentration	chronic	5	2	2
Feeling unmotivated	chronic	5	1	1
Bad social/emotional resonance	chronic	5	0	0
Depressed mood	chronic	4	1	0

1 stands for the lowest value and 10 for the highest; “chronic” means in partial remission or not remitted after beginning; “acute” means just now.

**Table 2:** BDI-II, BSCL (pre- vs. postintervention 1 vs. follow-up).

	Score (Pre-Intervention)	Score (Post-Intervention)	Score (6M-Follow-Up)
Total score BDI-II	13	3	1
Global Severity Index (BSCL)	62+	40	34−
Depressiveness (BSCL)	65+	57	49

BDI-II scores are raw data/scores. BSCL-scores are T-scores (M = 50, SD = 10) + = above the norm − = underneath the test norm.

## Data Availability

Not applicable.
